# Epidemiology of human infections with highly pathogenic avian influenza A(H7N9) virus in Guangdong, 2016 to 2017

**DOI:** 10.2807/1560-7917.ES.2017.22.27.30568

**Published:** 2017-07-06

**Authors:** Min Kang, Eric H Y Lau, Wenda Guan, Yuwei Yang, Tie Song, Benjamin J Cowling, Jie Wu, Malik Peiris, Jianfeng He, Chris Ka Pun Mok

**Affiliations:** 1 Guangdong Provincial Center for Disease Control and Prevention, Guangzhou, PR China; 2 MK, EYL and WG contributed equally to this work; 3 WHO Collaborating Centre for Infectious Disease Epidemiology and Control, School of Public Health, Li Ka Shing Faculty of Medicine, The University of Hong Kong, Hong Kong Special Administrative Region, PR China; 4 State Key Laboratory of Respiratory Disease, National Clinical Research Center for Respiratory Disease, First Affiliated Hospital of Guangzhou Medical University, Guangzhou, PR China; 5 HKU-Pasteur Research Pole, School of Public Health, HKU Li Ka Shing Faculty of Medicine, The University of Hong Kong, Hong Kong Special Administrative Region, PR China

**Keywords:** Influenza, H7N9, HPAI, Guangdong

## Abstract

We describe the epidemiology of highly pathogenic avian influenza (HPAI) A(H7N9) based on poultry market environmental surveillance and laboratory-confirmed human cases (n = 9) in Guangdong, China. We also compare the epidemiology between human cases of high- and low-pathogenic avian influenza A(H7N9) (n = 51) in Guangdong. Case fatality and severity were similar. Touching sick or dead poultry was the most important risk factor for HPAI A(H7N9) infections and should be highlighted for the control of future influenza A(H7N9) epidemics.

A novel avian influenza A(H7N9) virus emerged in mainland China in March 2013, and 1,533 human infections with 592 deaths have been reported to the World Health Organization as of June 2017 [1]. The virus was low-pathogenic to poultry but caused varying disease severity in humans and there was an increase in virus detection in poultry and humans during winter [2-4]. There was a surge of human disease in the winter and spring period of 2016/17 with more than 500 reported cases of influenza A(H7N9) [1], surpassing the maximum number (ca 300 cases) of the previous four epidemic waves [5]. The 2016/17 epidemic wave has extended to summer and not yet come to an end at the time of writing, much longer than the earlier epidemics. 

Before the 2016/17 winter wave, all influenza A(H7N9) viruses identified from poultry and humans were low-pathogenic avian influenza (LPAI). However, influenza A(H7N9) viruses isolated from two human cases in Guangdong in mid-February 2017 showed insertion of four amino acids at the cleavage site of the haemagglutinin protein, indicating that the virus had become highly pathogenic to chickens [6,7]. In this study, we report the prevalence of the highly pathogenic avian influenza (HPAI) A(H7N9) virus in Guangdong poultry markets through active surveillance and compare the epidemiological characteristics and clinical outcomes between the patients infected with the HPAI and LPAI A(H7N9) viruses in Guangdong province during the 2016/17 season.

## Environmental surveillance for influenza A(H7N9) virus and detection of HPAI and LPAI A(H7N9) human cases

Influenza A(H7N9) infection is a notifiable disease in mainland China. From 1 November 2016 through 31 March 2017, a total of 60 RT-PCR laboratory-confirmed human influenza A(H7N9) cases were detected and reported to the Guangdong Provincial Center for Disease Control and Prevention (Guangdong CDC). All influenza A(H7N9)-positive samples were sent to Guangdong CDC for further discrimination between HPAI and LPAI A(H7N9) viruses by sequencing the connecting peptide region of the haemagglutinin gene; nine HPAI and 51 LPAI A(H7N9) human cases were identified.

The H7 detection rate from environmental surveillance [8] in the Guangdong live poultry markets (LPMs) increased in December 2016, and there was a surge in the number of reported influenza A(H7N9) human cases starting in mid-December 2016 (Figure).

**Figure:**
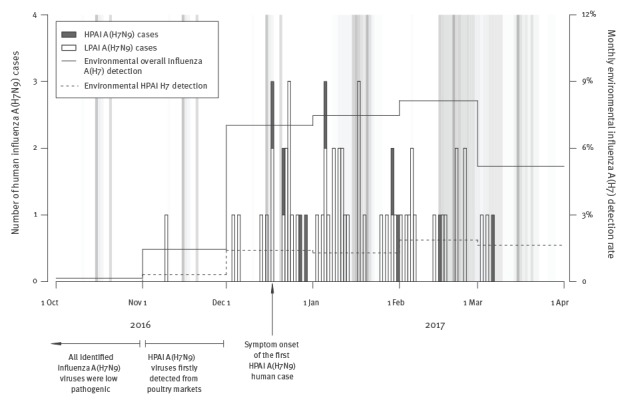
Epidemic curves for HPAI and LPAI A(H7N9) human cases, Guangdong province, 1 November 2016–31 March 2017 (n = 60)

HPAI: highly pathogenic avian influenza; LPAI: low pathogenic avian influenza.

Grey bars: time when rest days or closure in poultry markets were implemented. Darker bars: more cities implementing the market interventions on the same day. Dashed line: monthly environmental influenza A(H7) detection rate from 105 poultry markets in Guangdong province.

HPAI A(H7N9) viruses were first identified in poultry markets in four of 21 cities in Guangdong in November 2016; before that, only LPAI A(H7N9) viruses has been detected. The first patient infected with HPAI A(H7N9) viruses fell ill on 17 December 2016 (Figure) [9]. Genetic characterisation of HPAI A(H7N9) was carried out in retrospect and thus, LPAI and HPAI A(H7N9) patients were managed in the same way. Different cities in Guangdong have implemented routine rest days in live poultry markets since 2013 and temporary market closure for 3 to 14 days was also introduced in late February 2017, when the demand for chicken subsided after the Chinese New Year holiday and an increased number of human influenza A(H7N9) cases were reported in Guangdong and other provinces in China (Figure).

### Comparison of HPAI and LPAI A(H7N9) human cases in Guangdong

During the study period, influenza A(H7N9) human cases were detected in 16 of 21 cities in Guangdong (Table 1). 

**Table 1:** Geographical location of the HPAI and LPAI A(H7N9) human cases detected in Guangdong province, 1 November 2016–31 March 2017 (n = 60)

City^a^	HPAI A(H7N9) n = 9	LPAI A(H7N9) n = 51
Guangzhou	1	12
Shenzhen	0	6
Dongguan	0	4
Foshan	0	5
Zhanjiang	0	0
Jieyang	1	2
Maoming	0	0
Shantou	0	0
Huizhou	0	4
Jiangmen	0	5
Meizhou	0	2
Zhaoqing	2	1
Qingyuan	2	1
Zhongshan	1	3
Heyuan	1	0
Shanwei	0	1
Shaoguan	1	1
Chaozhou	0	3
Yangjiang	0	0
Yunfu	0	1
Zhuhai	0	0

HPAI: highly pathogenic avian influenza; LPAI: low-pathogenic avian influenza.

^a^ By descending order of population size.

We collected demographic, epidemiological and clinical data on influenza A(H7N9) cases using standardised forms. Collection and analyses of data from influenza A(H7N9) human cases were part of an ongoing public health investigation of emerging outbreaks and thus were exempt from institutional review board assessment in China [10]. Descriptive analyses were carried out and multivariable analysis was fitted to a limited set of exposure variables. 

We compared the demographic characteristics of the hospitalised human cases infected with HPAI A(H7N9) (n = 9) vs LPAI A(H7N9) virus (n = 51) (Table 2). We found a higher proportion of HPAI A(H7N9) cases who were female (HPAI: 55.6% vs LPAI: 25.5%, Fisher’s exact test p = 0.111) and a lower proportion living in areas with strictly regulated poultry trading, where live poultry sales were banned and only trading of dressed or chilled poultry is allowed (HPAI: 0.0% vs LPAI: 23.5%, Fisher’s exact test p = 0.182). The median age was similar for HPAI and LPAI A(H7N9) cases.

**Table 2:** Characteristics of laboratory-confirmed HPAI and LPAI A(H7N9) human cases detected in Guangdong province, 1 November 2016–31 March 2017 (n = 60)

Characteristics	HPAI A(H7N9) n = 9			LPAI A (H7N9) n = 51			p value^b^
	n	%	95% CI **^a^**	n	%	95% CI **^a^**	
Median age (in years with IQR)	57 years		45–63	56 years		49–67	0.780
Male sex	4	44	14–79	38/51	75	60–86	0.111
Poultry worker	0	0	0–34	3/51	6	1–16	1.000
Residence in area with strictly regulated poultry trading	0	0	0–34	12/51	24	13–37	0.182
Exposure to live poultry^c^							
Any exposure to live poultry	7	78	40–97	30/51	59	44–72	0.460
Touched live poultry	7	78	40–97	15/50	30	18–45	0.010
Bought live poultry	0	0	0–34	15/50	30	18–45	0.095
Prepared live poultry	3	33	7–70	9/50	18	9–31	0.369
Consumed live poultry	4	44	14–79	12/50	24	13–38	0.236
Raising backyard poultry	7	78	40–97	15/51	29	17–44	0.009
Exposure to sick or dead poultry^c^							
Any exposure to sick or dead poultry	6	67	30–93	5/50	10	3–22	0.001
Within 1 m of sick of dead poultry	1	11	0–48	0/50	0	0–7	0.153
Touched sick or dead poultry	5	56	21–86	5/50	10	3–22	0.005
Consumed sick or dead poultry	1	11	0–48	0/50	0	0–7	0.153
Exposure to poultry markets^c^							
Visited retail LPM	5	56	21–86	31/50	62	47–75	0.726
Visited wholesale LPM	1	11	0–48	9/48	19	9–33	1.000
Visited dressed poultry market	0	0	0–34	1/48	2	0–11	1.000
Symptoms							
Fever	8	89	52–100	47/51	92	81–98	0.570
Cough	8	89	52–100	44/51	86	74–94	1.000
Sore throat	1	11	0–48	13/51	26	14–40	0.671
Weakness	6	67	30–93	19/51	37	24–52	0.145
Muscle pain	4	44	14–79	10/51	20	10–33	0.193
Shortness of breath	0	0	0–34	6/51	12	4–24	0.578
Diarrhoea	0	0	0–34	2/51	4	0–13	1.000
Underlying conditions	2	22	3–60	29/51	57	42–71	0.076
Pneumonia	9	100	66–100	51/51	100	93–100	1.000
ICU admission	8	89	52–100	45/51	88	76–96	1.000
Died	5	56	21–86	22/51	43	29–58	0.718
Median incubation period^d^ (in days with 95% CI)	5.2		2.8–9.7	3.8		3.0–4.6	0.619
Median durations (in days with IQR)							
Onset to laboratory confirmation	8.0		6.0–11.0	8.0		6.0–10.0	0.700
Onset to hospitalisation	3.0		1.0–5.0	4.0		3.0–5.0	0.451
Hospitalisation to ICU admission	2.0		1.8–2.0	1.0		1.0–2.0	0.052
Hospitalisation to death	28.0		3.0–30.0	8.0		5.3–11.5	0.434
Hospitalisation to discharge	29.0		26.8–37.0	20.0		14.8–26.0	0.049

CI: confidence interval; HPAI: highly pathogenic avian influenza; ICU: intensive care unit; IQR: interquartile range; LPAI: low-pathogenic avian influenza; LPM: live poultry market.

**^a^** CIs shown for percentages unless otherwise indicated.

^b^ Fisher’s exact test for categorical variables, Wilcoxon signed rank test for continuous variables, and likelihood ratio test under accelerated failure time model for incubation periods.

^c^ In the 10 days before symptom onset.

^d^ Based on log-normal distribution, accounting for interval censoring of poultry or LPM exposure.

The clinical presentation was similar and all reported influenza A(H7N9) cases were severe and required hospitalisation (Table 2). However, fewer HPAI than LPAI A(H7N9) cases had underlying conditions (Fisher’s exact test p = 0.076). Significantly more HPAI A(H7N9) cases (77.8%) raised backyard poultry compared with LPAI A(H7N9) cases (29.4%; Fisher’s exact test p = 0.009). HPAI A(H7N9) cases were associated with touching live or dead poultry (Fisher’s exact test p = 0.010 and 0.005 respectively), while exposures to poultry markets were similar. By including touching live poultry and touching dead poultry respectively in two multivariable ridge logistic regression models along with raising backyard poultry, these exposures remained independently significant, and touching sick or dead poultry was the single most important risk factor for HPAI A(H7N9) infections (Table 3).

**Table 3:** Multivariable logistic ridge regression analysis of risk factors for HPAI compared with LPAI A(H7N9) human infections (n = 60)

Exposure	HPAI A(H7N9) infections AOR (95% CI)	
	Model 1	Model 2
Raise backyard poultry	2.13 (1.02–6.06)	1.97 (1.01–6.33)
Touched live poultry	2.11 (1.01–5.76)	Not entered
Touched sick or dead poultry	Not entered	5.35 (1.09–32.60)

AOR: adjusted odds ratio; CI: confidence interval; HPAI: highly pathogenic avian influenza; LPAI: low-pathogenic avian influenza.

Fatality risks among hospitalised patients were similar and remained high (40–60%) for both LPAI and HPAI A(H7N9) cases. Duration from hospitalisation to admission to an intensive care unit (ICU) (p = 0.052) and from hospitalisation to discharge (p = 0.049) were significantly longer for HPAI A(H7N9) patients. Among patients who died, more HPAI A(H7N9) patients had duration from hospitalisation to death longer than 21 days (HPAI: 3/5 vs LPAI: 2/22; p-value = 0.030).

## Discussion

In this study, we described the prevalence of influenza A(H7N9) viruses in Guangdong poultry markets during the 2016/17 season and compared the demographical, epidemiological and basic clinical characteristics between the HPAI and LPAI A(H7N9) human cases. Since the end of the study period, two more influenza A(H7N9) cases have been identified in Guangdong but have yet to be characterised as HPAI or LPAI. Although the study was limited by a relatively small sample with 9 HPAI and 51 LPAI A(H7N9) cases, comparing HPAI and LPAI A(H7N9) patients within Guangdong province provided a direct comparison by reducing potential impact of geographical heterogeneity in community exposure patterns to poultry, access to healthcare and clinical management [11].

We identified touching sick or dead poultry as the single most important risk factor for HPAI A(H7N9) human infections, followed by raising backyard poultry and touching live poultry. Similar peptide sequences at the multiple basic amino acid cleavage site of HA have been reported for H7 viruses in Chile and Canada that were shown to be highly pathogenic in chickens by intravenous pathogenicity tests [12]. Moreover, a recent study demonstrated that the insertion of the four amino acids in this region enabled trypsin-independent infectivity of this virus [13]. This observation strongly implicates that the new identified HPAI A (H7N9) virus has a high virulence phenotype in chicken. In general, HPAI viruses in poultry disseminate to multiple organs including muscle, whereas LPAI viruses are restricted to the respiratory and gastrointestinal tracts. Thus, HPAI A(H7N9) virus-infected poultry carcasses may be more infectious to humans than poultry infected with LPAI A(H7N9). These findings support our analysis that the major route of transmitting the HPAI A(H7N9) virus to humans will be through the contact of sick or dead rather than healthy poultry. However, the pathogenicity of chickens with HPAI A(H7N9) infection needs to be investigated urgently. 

Clinical outcomes were comparable between hospitalised HPAI and LPAI A(H7N9) cases, except that the time from hospitalisation to discharge were longer for HPAI A(H7N9) cases. The importance of avoiding touching sick and dying poultry should be highlighted to the public for the control of HPAI A(H7N9). Among those raising backyard poultry, 10 of 22 reported touching sick or dead poultry, while none reported touching sick or dead poultry among the 38 not raising backyard poultry. Strengthening awareness of the risks of backyard poultry will be crucial in the control of future HPAI A(H7N9) epidemics, especially since recent A(H7N9) cases have shifted to rural areas where backyard poultry is more prevalent [5].

We observed longer average hospital stays for HPAI A(H7N9) patients although there was no difference in clinical progression and outcome between HPAI and LPAI A(H7N9) patients. However, since there was no statistically significant difference in admission to ICU and clinical outcomes between HPAI and LPAI A(H7N9) patients, we still cannot determine whether the HPAI A(H7N9) viruses caused higher severity in patients. That LPAI A(H7N9) patients had a shorter duration from hospitalisation to ICU admission and a smaller proportion with long duration from hospitalisation to death may be partially explained by the higher frequency of underlying conditions in this group. Detailed clinical investigation of virus shedding, virus dissemination and the levels of inflammation may be able to shed light on these questions.

Our analysis only focused on the comparison among severe influenza A(H7N9) patients. In earlier epidemics, a spectrum of cases from of asymptomatic to mild and severe was detected in Guangdong [3], however in the more recent epidemic waves in China, the detected cases tended to be more severe [11]. Therefore, our understanding of the full range of severities of the HPAI A(H7N9) infections is still incomplete.
